# Supplementary material for: T cell-independent eradication of experimental glioma by intravenous TLR7/8-agonist-loaded nanoparticles
Source: Nat Commun. 2023 Feb 11;14:771. doi: 10.1038/s41467-023-36321-6 (PMC9922247; doi:10.1038/s41467-023-36321-6)
Supplement: Supplementary file 1 — Supplementary information [file 41467_2023_36321_MOESM1_ESM.docx]

- **Supplementary information -**

**T cell-independent eradication of experimental glioma by intravenous TLR7/8-agonist-loaded nanoparticles**

Verena Turco^1,2,3^, Kira Pfleiderer^1,3^, Jessica Hunger^1,3,4^, Natalie K. Horvat^4,5,6^, Kianush Karimian-Jazi^3^, Katharina Schregel^3^, Manuel Fischer^3^, Gianluca Brugnara^3^, Kristine Jähne^1,2^, Volker Sturm^3^, Yannik Streibel^3^, Duy Nguyen^7^, Sandro Altamura^,5,6^, Dennis A. Agardy^1,2,4^, Shreya S. Soni^8^, Abdulrahman Alsasa^8^, Theresa Bunse^1,2^, Matthias Schlesner^7,9^, Martina U. Muckenthaler^5,6^, Ralph Weissleder^10,11^, Wolfgang Wick^12,13^, Sabine Heiland^3^, Philipp Vollmuth^3^, Martin Bendszus^3^, Christopher B. Rodell^8^, Michael O. Breckwoldt^1,3,§,#^ and Michael Platten^1,2,§,#^

**Supplementary Figures**


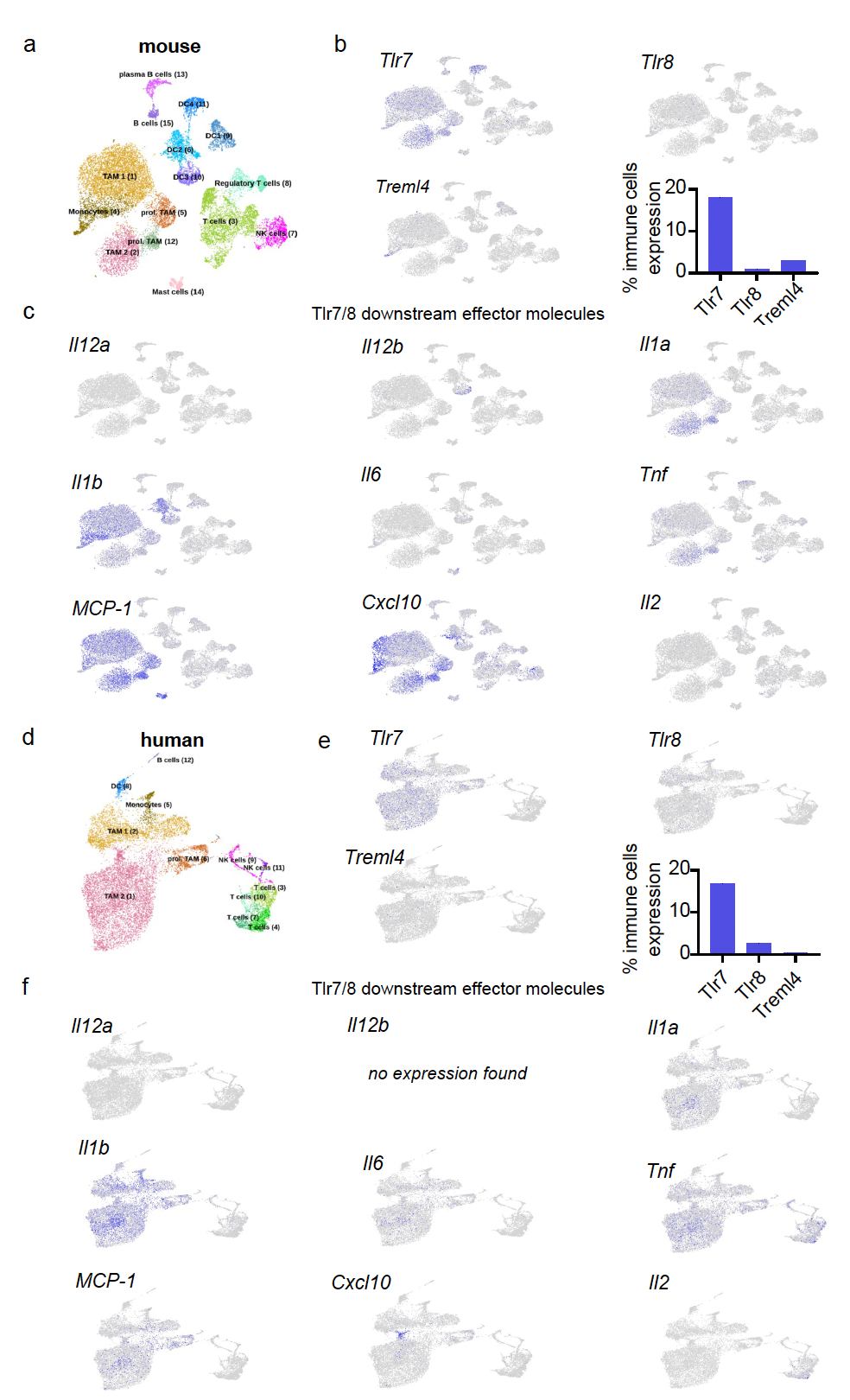


**Suppl. Fig. 1: TLR 7 expression and downstream pathways as assessed by single-cell RNA sequencing**

**a-f:** Analysis of single-cell RNA sequencing data of tumor associated myeloid cells (TAMs) of Gl261-bearing mice and human glioma based on the previously published dataset by Pombo Antunes et al.^1^ Landscape of TAMs of Gl261-bearing mice (**a**) and glioma patients (**d**). Single-cell expression and quantification of TLR7, TLR8 and Treml4 on TAMs of Gl261-bearing mice (**b**) and glioma patients (**e**). Single-cell expression analysis of downstream effector molecules of the TLR7/8 pathway on Gl261 associated myeloid cells (**c**) and myeloid cells of glioma patients (**f**).


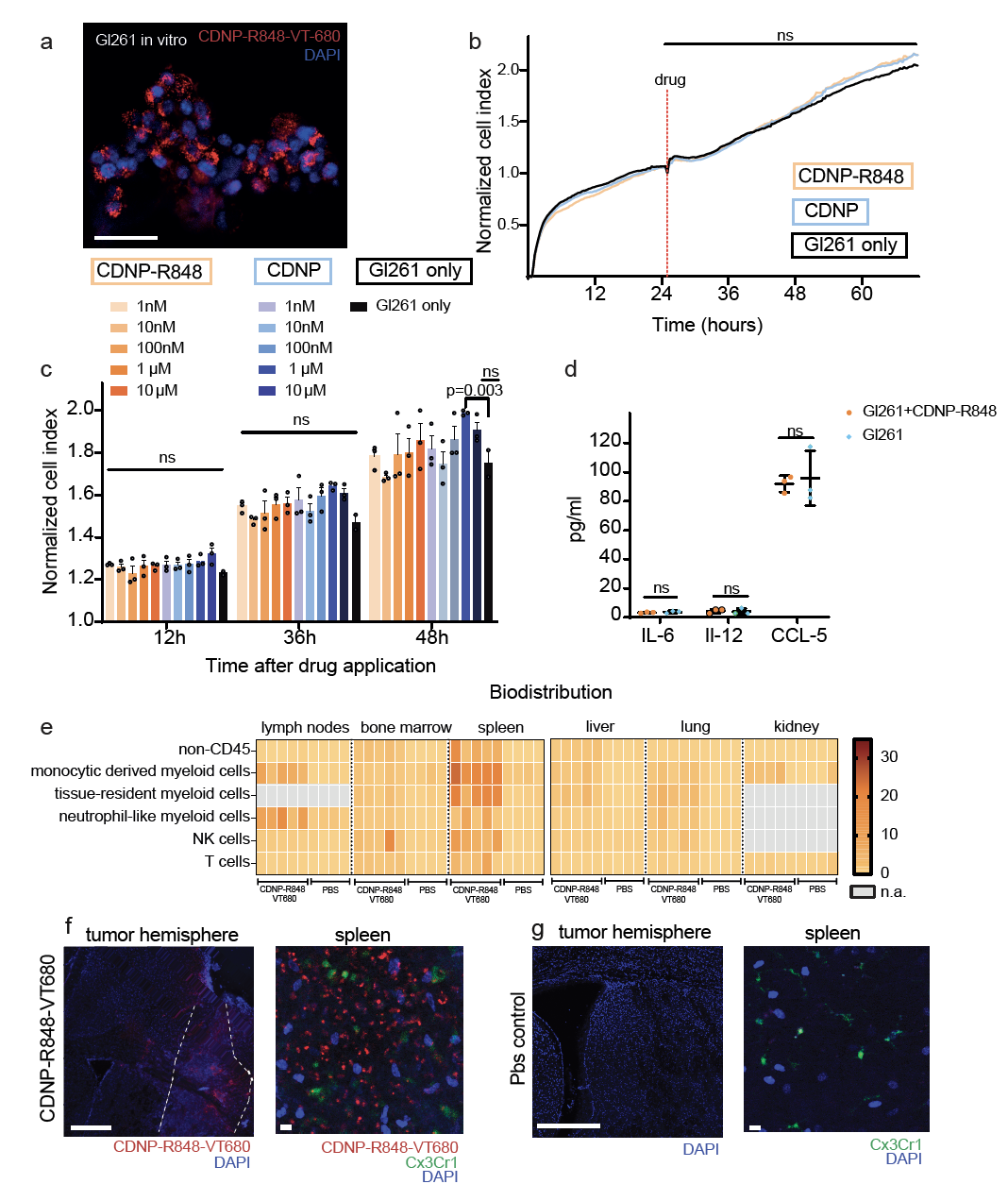


**Suppl. Fig 2: Assessing CDNP-R848 effects on Gl261 glioma cells *in vitro* and biodistribution of CDNP-R848 in vivo**

**a:** Immunofluorescence imaging of Gl261 tumor cells seeded in cell culture medium supplemented with CDNP-R848-VT680 overnight show avid NP uptake. **b-c:** Proliferation of Gl261 tumor cells treated with increasing amounts of CDNP-R848 or CDNP vehicle control (1nM to 10µM) was assessed by RTCA assay. n=3 biological replicates from one independent experiment **d:** Cultured Gl261 tumor cells were treated with 1µM CDNP-R848 or vehicle and chemokine and cytokine production was quantified in supernatants by ELISA. All other tested cytokines were below the detection limit. n=3 biological replicates from one independent experiment **e**: Biodistribution analysis of CDNP-R848-VT680 within immune cells 24h after a single, intravenous dose on day 16 as assessed by flow cytometry of monocyte derived myeloid cells (CD45^+^ CD11b^+^, Ly6g^-^, Ly6c^+^), tissue resident myeloid cells (CD45^+^, CD11b^+^, Ly6g^-^, F4/80^+^), neutrophil like myeloid cells (CD45^+^, CD11b^+^, Ly6g^+^), NK cells (CD45^+^, CD3^-^ NK1.1^+^), T-cells (CD45^+^, CD3^+^) and microglia (CD45^+^ CD11b^intermediate^) in lymphnodes, bone marrow, spleen, liver, lung and kidney. n=5 CDNP-R848-VT680 mice and 4 pbs treated animals. **f-g**: C57Bl/6J mice were treated with 100 µl CDNP-R848-VT680 or PBS control after intracranial Gl261 tumor injection. NP distribution in brain and spleen in CDNP-R848-VT680 injected animals (f) vs PBS injected control (g). CDNP-R848-VT680 accumulates specifically in the glioma region (dashed line). Images are representative of n=3 mice. Scale bar in a is 50µm, in f,g 500µm for tumor hemispheres and 20µm for spleens. Data is from one independent experiment and presented as mean ± SEM. Statistical significance was determined by one-way ANOVA or by two-tailed Student’s test.


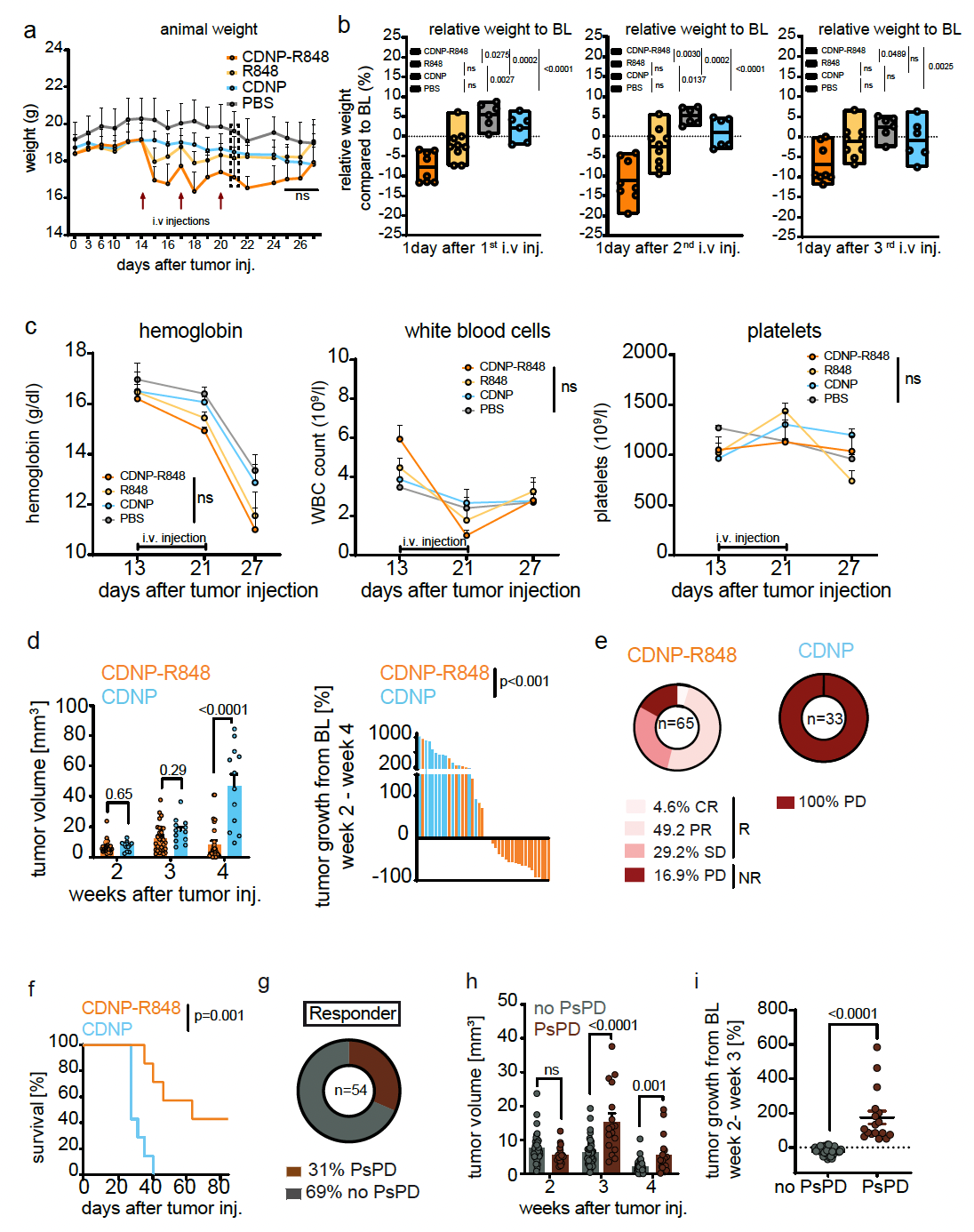


**Suppl. Fig. 3 Organ specific toxicity assessment, efficacy of CDNP-R848 and PsPD**

**a:** C57Bl/6J mice bearing intracranial Gl261 tumors were treated intravenously with 100 µl CDNP-R848, R848, CDNP or PBS on day 14, day 17 and day 20 after baseline MRI and randomization on day 13. Mice were monitored by serial MR imaging on day 19 and day 25. Weight analysis during the course of the experiment is shown in a. In panels a to c: n=8 mice in the CDNP-R848 group, n=9 for R848, n=6 for CDNP and PBS treated mice from independent experiment. **b:** Relative weight loss compared to baseline weight of CDNP-R848, R848, CDNP and PBS treated mice 1 day after respective intravenous therapy administration. Box plots indicate min / max and line at mean **c:** Serial blood analysis was performed by taking cheek blood on day 13 (baseline) and day 21 after completion of therapy. Terminal heart blood was taken on day 27. Hemoglobin, platelets and white blood cell count were assessed. **d**: tumor volumes and waterfall plot of CDNP-R848 *vs* CDNP vehicle treated mice (pooled data from n=12 mice for CDNP group and 28 mice for CDNP-R848 from 4 independent experiments) **e**: Overall response rate towards CDNP-R848 and CDNP. R, response; NR, non-response; PD, progressive disease. Response rates were calculated based on Aslan et al^2^. Pooled data from all experiments included in this study (n=65 mice for CDNP-R848 and 33 mice for CDNP group from 9 independent experiments). **f**: survival analysis of CDNP-R848 and CDNP vehicle treated mice (n=7 mice per group, one independent experiment). **g-i**: tumor volumes and tumor growth in week 3 compared to baseline of week 2 of responder mice that showed PsPD (31%, n=17 mice) vs. mice that did not show PsPD (69%, n=37 mice, pooled data from for independent experiments) after CDNP-R848 treatment. Data are presented as mean ± SEM. Statistical significance was determined by unpaired two-tailed Student t-tests or one-way ANOVA with Tukey’s test and mixed-effect analysis with Turkey’s test.


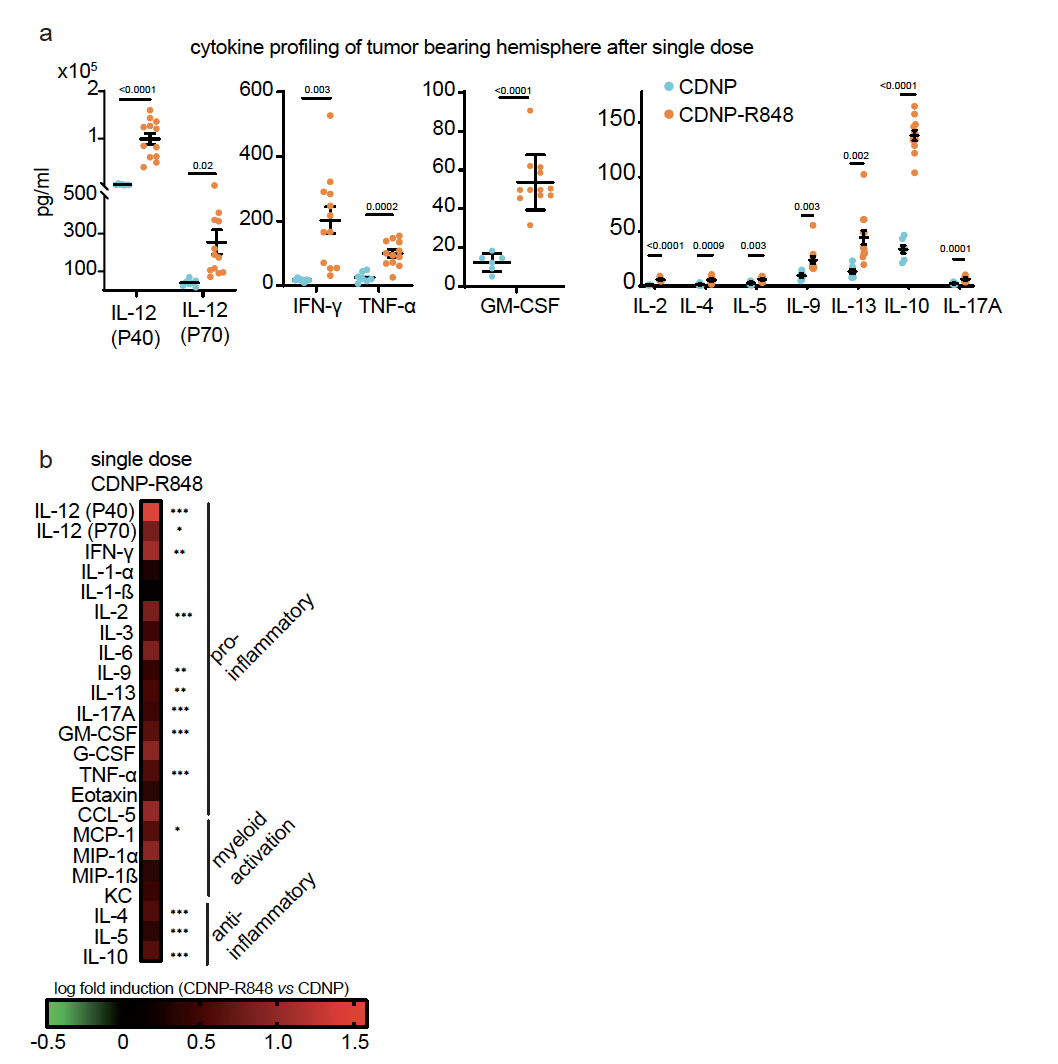


**Suppl. Fig. 4** **Cytokine profiling after a single dose of CDNP-R848 in the tumor bearing hemisphere of Gl261 glioma mice *in vivo***

**a:** C57Bl/6J mice were treated with a single dose of CDNP-R848 (n=12 mice) or CDNP vehicle control (n=7 mice) and terminated 48 to 72 hours after drug treatment. Data is pooled from two independent experiments. After enzymatic digestion of tumor-bearing hemisphere, supernatant was taken and Luminex cytokine profiling was performed for indicated cytokines. **b**: Heatmap shows Luminex cytokine results after a single intravenous dose of CDNP-R848 or CDNP. All data are presented as individual values and the mean ± SEM. Statistical significance was determined by two-tailed student’s test.

**
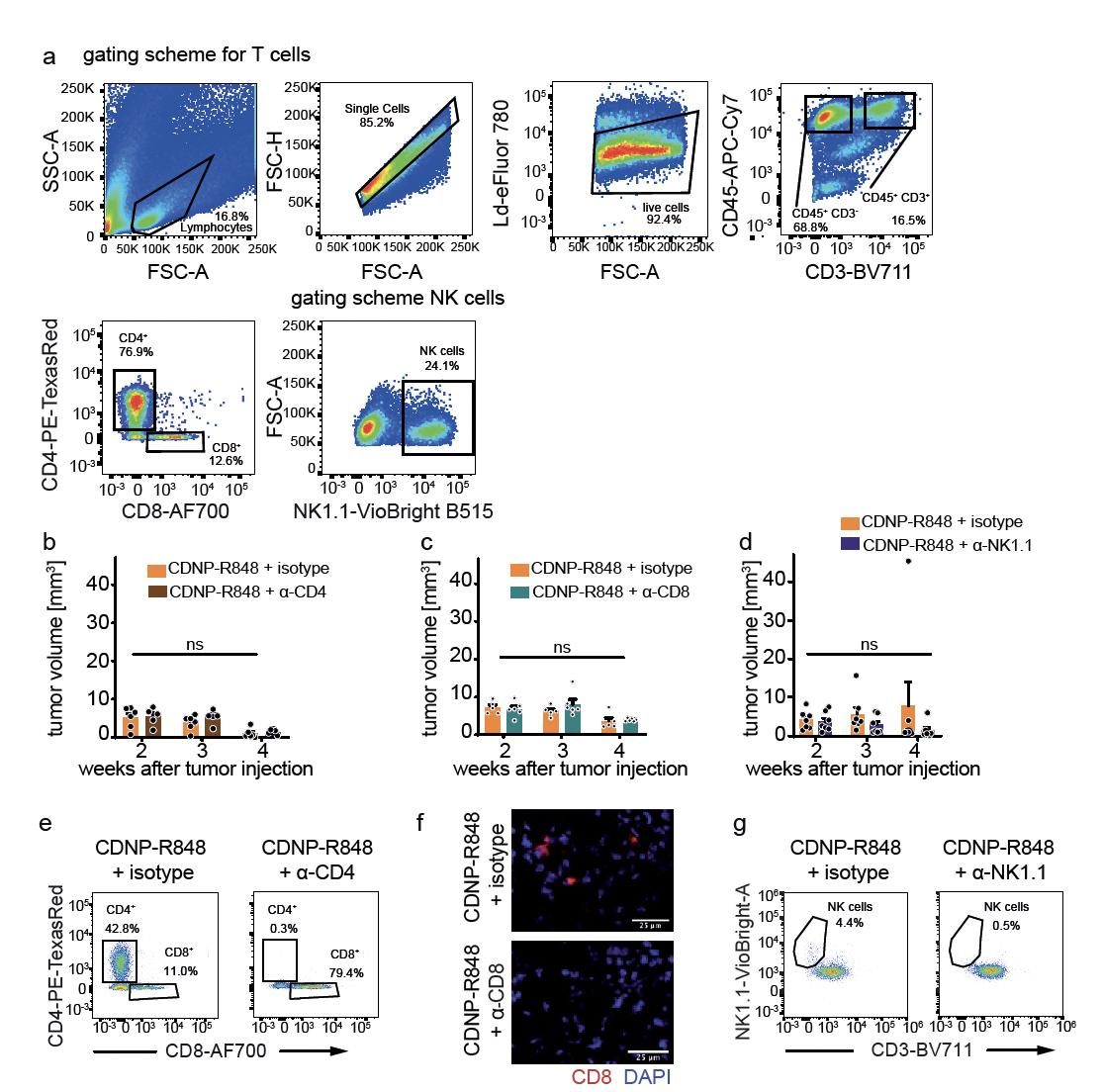
Suppl. Fig. 5: Depletion of CD4, CD8 and NK cells does not abrogate CDNP-R848 treatment efficacy**

**a:** Gating strategy for NK and T cells used Figure 3c-h and in Suppl. Figure 5e,g**. b-d**: Tumor volumes in CDNP-R848 treated mice that had received α-CD8 (n=6 mice), α-CD4 (n=5 mice), α-NK1.1 (n=7) or isotype (n=18 mice) to deplete CD8 T cells, CD4 T cells or NK cells in comparison to isotype treated controls**. e-g:** Depletion was confirmed at day 27 in the TME by flow cytometry (**e,g**) or by immunofluorescence microscopy (**f**). Immunofluorescent images are representative images from n=6 mice per group. All data are presented as individual values and the mean ± SEM. Statistical significance was determined by two-tailed student’s test.

**
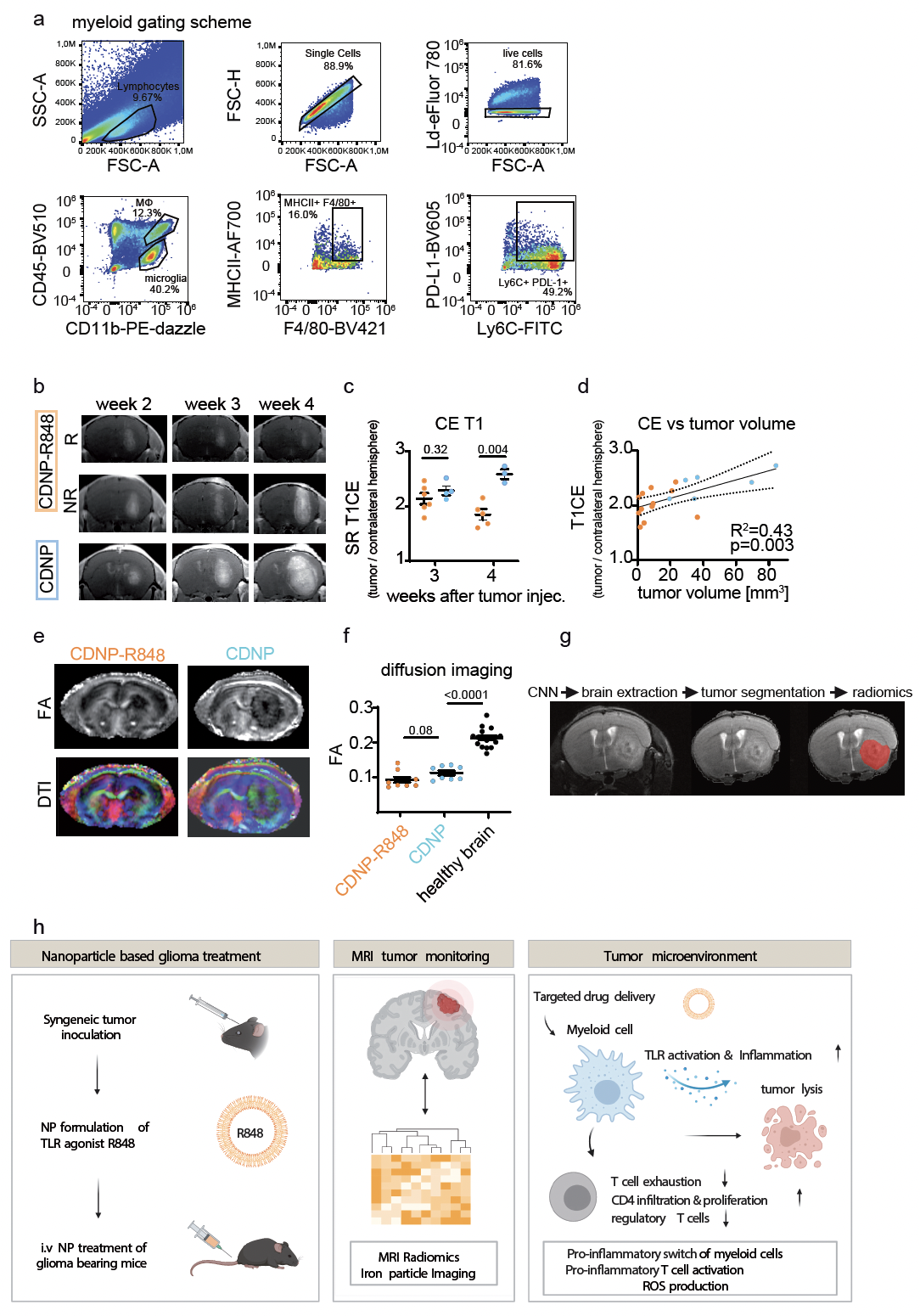
**

**Suppl. Fig. 6**: **Imaging markers of response to CDNP-R848 and proposed mode of action**

**a**: Gating strategy for flow cytometric quantification of macrophages (CD45^high^, CD11b^+^) and microglia (CD45^intermediate^, CD11b^+^) and functional phenotyping by proinflammatory (MHC-II^+^, F4/80^+^) and anti-inflammatory (PD-L1^+^, Ly6C^+^) markers. This gating strategy was used in Figure 5 a-f **b**: Representative T1-w Gd-contrast enhanced MRI showing response (R) and resistance (non-response, NR) during CDNP-R848 treatment compared to CDNP vehicle. **c**: Quantification of signal ratio (SR) T1-w contrast enhanced (CE) imaging. **d**: Correlation analysis of Gd-contrast enhancement and tumor size. n=5 mice for CDNP-R848 and n=4 mice for CDNP from one independent experiment **e:** MR diffusion imaging at week 3 in the CDNP-R848 and CDNP vehicle group. Representative fractional anisotropy (FA) and diffusion tensor images (DTI) are shown **f:** Quantification of FA in the tumor core after CDNP-R848 (n=8 mice) vs CDNP control treatment (n=8 mice) and healthy contralateral hemisphere. Data is pooled from two independent experiments **g:** A computational neuronal network (CNN) was developed for automated brain extraction, tumor segmentation and radiomic feature analysis. **h**: Graphical summary and proposed response mechanism to CDNP-R848 in the glioma TME. Panel h was created with BioRender.com. All data are presented as individual values and the mean ± SEM. Correlation analysis were performed by Spearman correlation. Statistical significance was determined by two-tailed student’s test.

**Supplementary Tables**

**Suppl. Table 1 Antibodies used for *in vivo* cell depletion**

| Antibody | LOT | Clone | Manufacturer | Catalogue # | Dilution |
| --- | --- | --- | --- | --- | --- |
| mCD8a | 624616A2 | 2.43 | BioXCell | BE0061 | 500µg /mouse in 200µl PBS |
| mNK1.1 | 735620O1 | PK136 | BioXCell | BE0036 | 200µg /mouse in 200µl PBS |
| mCD4 | 699918O1B | GK1.5 | BioXCell | BE0003-1 | 1000µg/mouse in 200µl PBS |

**Suppl. Table S2 Antibodies used for Flow Cytometry**

| Antibody | Fluorophore | Clone | Manufacturer | Dilution |
| --- | --- | --- | --- | --- |
| CD38 | FITC | 90 | BioLegend | 1:200 |
| CD86 | PE | GL-1 | BioLegend | 1:400 |
| CD64 | PerCP-eFluor 710 | X54-5/7.1 | ThermoFisher | 1:400 |
| MHC II | PE-Cy7 | M5/114.15.2 | BioLegend | 1:100 |
| MerTK | Brilliant Violet 421 | 108928 | BD Biosciences | 1:100 |
| CD172 | APC/Cy7 | P84 | BioLegend | 1:800 |
| CD71 | Brilliant Violet 510 | RI7217 | BioLegend | 1:200 |
| mCD45 | BV510, 30 | F11 | BioLegend | 1:100 |
| mCD3 | BV711 | 17A2 | BioLegend | 1:100 |
| CD38 | FITC | 90 | BioLegend | 1:100 |
| mCD11b | PE-dazzle | M1/70 | BioLegend | 1:100 |
| mCD4 PE | Texas Red | RM4-5 | Invitrogen | 1:100 |
| mCD8 | AF700 | 53-6.7 | BioLegend | 1:133 |
| mF4/80 | BV421 | BM8 | BioLegend | 1:100 |
| mMHCII | AF700 | M5/114.15.2 | BioLegend | 1:100 |
| mPD-L1 | BV605 | 10F.9G2 | BioLegend | 1:100 |
| mLy6C | FITC | HK1.4 | BioLegend | 1:100 |
| mLy6C | APC | HK1.4, | BioLegend | 1:100 |
| mFoxP3 | FITC | FJK-16s | Invitrogen | 1:133 |
| mLag3 | PE-Cy7 | eBioC9B7W | Invitrogen | 1:100 |
| mCD45 | APC-Cy7 | 30-F11 | BD Biosciences | 1:100 |
| mCD25, | PerCPCy5.5 | PC61 | BioLegend, | 1:100 |
| mPD-L1 | BV711 | 10F.9G2 | BioLegend | 1:100 |
| mPD-1 | BV421 | 29F.1.A12 | BioLegend | 1:100 |
| mKi67 | eFlour450 | SolA15 | Invitrogen | 1:50 |
| mNK1.1 | Vio Bright B515 | REA1162 | Miltenyi Biotec | 1:100 |
| mCD16/CD32 | n/a | 93 | eBioscience | 1:100 |
| Live / dead | eFluor 780 | n/a | eBioscience | 1:100 |

**Suppl. Table 3 Primers for quantitative RT-PCR *(mus musculus)***

| Gene | Sequence |
| --- | --- |
| *Il-1β* | Forward 5' GCAACTGTTCCTGAACTCAACT 3' |
|  | Reverse 5' ATCTTTTGGGGTCCGTCAACT 3' |
| *Il-6* | Forward 5' GCTACCAAACTGGATATAATCAGGA 3' |
|  | Reverse 5' CCAGGTAGCTATGGTACTCCAGAA 3' |
| *Il-12* | Forward 5' CCATCAGCAGATCATTCTAGA 3' |
|  | Reverse 5' CGCCATTATGATTCAGAGACTG 3' |
| *Lcn2* | Forward 5' CCATCTATGAGCTACAAGAGAACAAT 3' |
|  | Reverse 5' TCTGATCCAGTAGCGACAGC 3' |
| *Nos2* | Forward 5' TGGAGACTGTCCCAGCAATG 3' |
|  | Reverse 5' CAAGGCCAAACACAGCATACC 3' |
| *Rpl19* | Forward 5' AGGCATATGGGCATAGGGAAGAG 3' |
|  | Reverse 5' TTGACCTTCAGGTACAGGCTGTG 3' |
| *Tnfα* | Forward 5' TGCCTATGTCTCAGCCTCTTC 3' |
|  | Reverse 5' GAGGCCATTTGGGAACTTCT 3' |

**Supplementary References**

1. Antunes, A. R. P. *et al.* Single-cell profiling of myeloid cells in glioblastoma across species and disease stage reveals macrophage competition and specialization. *Nature Neuroscience* 24, 595–610 (2021).

2. Aslan, K. *et al.* Heterogeneity of response to immune checkpoint blockade in hypermutated experimental gliomas. *Nature Communications* 1–14 (2020) doi:10.1038/s41467-020-14642-0.
