# Supplementary material for: T cell-independent eradication of experimental glioma by intravenous TLR7/8-agonist-loaded nanoparticles
Source: Nat Commun. 2023 Feb 11;14:771. doi: 10.1038/s41467-023-36321-6 (PMC9922247; doi:10.1038/s41467-023-36321-6)
Supplement: Supplementary file 3 — Reporting Summary [file 41467_2023_36321_MOESM3_ESM.pdf]

nature portfolio

Corresponding author(s): Breckwoldt Platten

Last updated by author(s): Jan 12, 2023

# Reporting Summary

Nature Portfolio wishes to improve the reproducibility of the work that we publish. This form provides structure for consistency and transparency in reporting. For further information on Nature Portfolio policies, see our Editorial Policies and the Editorial Policy Checklist.

## Statistics

For all statistical analyses, confirm that the following items are present in the figure legend, table legend, main text, or Methods section.

| n/a | Confirmed | |
|---|---|---|
| ☐ | ☒ | The exact sample size (*n*) for each experimental group/condition, given as a discrete number and unit of measurement |
| ☐ | ☒ | A statement on whether measurements were taken from distinct samples or whether the same sample was measured repeatedly |
| ☐ | ☒ | The statistical test(s) used AND whether they are one- or two-sided<br>*Only common tests should be described solely by name; describe more complex techniques in the Methods section.* |
| ☒ | ☐ | A description of all covariates tested |
| ☐ | ☒ | A description of any assumptions or corrections, such as tests of normality and adjustment for multiple comparisons |
| ☐ | ☒ | A full description of the statistical parameters including central tendency (e.g. means) or other basic estimates (e.g. regression coefficient) AND variation (e.g. standard deviation) or associated estimates of uncertainty (e.g. confidence intervals) |
| ☐ | ☒ | For null hypothesis testing, the test statistic (e.g. *F*, *t*, *r*) with confidence intervals, effect sizes, degrees of freedom and *P* value noted<br>*Give P values as exact values whenever suitable.* |
| ☒ | ☐ | For Bayesian analysis, information on the choice of priors and Markov chain Monte Carlo settings |
| ☒ | ☐ | For hierarchical and complex designs, identification of the appropriate level for tests and full reporting of outcomes |
| ☒ | ☐ | Estimates of effect sizes (e.g. Cohen's *d*, Pearson's *r*), indicating how they were calculated |

*Our web collection on statistics for biologists contains articles on many of the points above.*

## Software and code

Policy information about availability of computer code

| | |
|---|---|
| Data collection | Flow cytometry data was acquired using the BD FACSDIVA software V.9.0 or Attune NxT Software V2.5. Microscopy data was collected using the ZEISS ZEN software V2.3. Multiparametric MR imaging was performed on a 9.4 Tesla horizontal bore small animal NMR scanner. |
| Data analysis | Flow cytometry data was analyzed using FlowJo V9 or V10. Analysis of microscopy images was done using FIJI/ImageJ V2.0. MRI data was analysed with Bruker's ParaVision Software 6.0, 3D Slicer V. 4.11.0 (www.slicer.org), FIJI/ImageJ V.2.0 imaging software, or MATLAB R2020a. Tumor volumes from MRI T2w images were calculated after semi-automatical segmentation of tumor areas using Osirix (Pixmeo) V. 4.12 , ImageJ or Amira imaging software (FEI) V.5.4. Radiomic features were calculated using medical imaging interaction toolkit (www.mitk.org; https://phabricator.mitk.org/w/mitk/changelog/release-v2022.10).The CNN for radiomic response prediction is available at github.com (https://github.com/NeuroAI-HD/HD-GLIOMOUSE). Prediction models were developed using R (Version 4.0.3, Foundation for Statistical Computing, Vienna). Full codes of all R scripts are available on request. Bio-Plex 200 System (Bio-Rad) was used for cytokine analysis and analyzed using Bio-Plex Manager software (version 6.0). Cell index (CI) for real-time dynamic cytotoxicity assessment was calculated within the RTCA Software Package version 1.2.1. All other statistics were done using GraphPad prism 8.0 |

For manuscripts utilizing custom algorithms or software that are central to the research but not yet described in published literature, software must be made available to editors and reviewers. We strongly encourage code deposition in a community repository (e.g. GitHub). See the Nature Portfolio guidelines for submitting code & software for further information.

## Data

Policy information about availability of data

All manuscripts must include a data availability statement. This statement should provide the following information, where applicable:

- Accession codes, unique identifiers, or web links for publicly available datasets
- A description of any restrictions on data availability
- For clinical datasets or third party data, please ensure that the statement adheres to our policy

All data sets generated or analysed during this study are included in this published article (and its supplementary information files). Custom written code is publicly available: The CNN for radiomic response prediction is available at https://github.com/NeuroAI-HD/HD-GLIOMOUSE. The radiomic brain extraction was performed with MITK, https://phabricator.mitk.org/w/mitk/changelog/release-v2022.10.

## Human research participants

Policy information about studies involving human research participants and Sex and Gender in Research.

| | |
|---|---|
| Reporting on sex and gender | n/a |
| Population characteristics | n/a |
| Recruitment | n/a |
| Ethics oversight | n/a |

Note that full information on the approval of the study protocol must also be provided in the manuscript.

# Field-specific reporting

Please select the one below that is the best fit for your research. If you are not sure, read the appropriate sections before making your selection.

☒ Life sciences ☐ Behavioural & social sciences ☐ Ecological, evolutionary & environmental sciences

For a reference copy of the document with all sections, see nature.com/documents/nr-reporting-summary-flat.pdf

# Life sciences study design

All studies must disclose on these points even when the disclosure is negative.

| | |
|---|---|
| Sample size | Sample size was calculated with the help of a biostatistician using R version 4.1.0 and as previously descrived (Aslan et al. Nat Comm., 2020). Assumptions for power analysis were as follows: alpha error: 5%; beta error: 20%. Values for standard deviation and differences between experimental groups were based on previous experiments. (whenever a similar data type was available). In all other cases a pilot group size was used. |
| Data exclusions | In case animals had to be sacrificed prior to the pre-defined endpoint (due to weight loss or other termination criteria), they were excluded from any downstream analysis. |
| Replication | Key experiments (response to CDNP-R848, tumor microenvironment phenotyping by flow cytometry) were all performed at least twice and data from one representative experiment or pooled data are shown in this manuscript as indicated in the figure captions. Replication of experiments were successfull and showed comparable results. All other experiments were performed once with biological replicates (as specified in figure legend). |
| Randomization | Mice were matched into the groups according to tumor size (measured by MRI) at the time of treatment start so ensure equal tumor volumes in the different treatmet arms. Tumor volumes at baseline were measured in a standardized manner before randomization was performed |
| Blinding | Intracranial tumor and in vitro BMDM experiments were performed in a blinded manner so the experimentation and analysis were blinded for treatment arm (MRI tumor volume assessment, flow cytometric analyses, qRT-PCR, histology). |

# Reporting for specific materials, systems and methods

We require information from authors about some types of materials, experimental systems and methods used in many studies. Here, indicate whether each material, system or method listed is relevant to your study. If you are not sure if a list item applies to your research, read the appropriate section before selecting a response.

## Materials & experimental systems

| n/a | Involved in the study |
|---|---|
| ☐ | ☒ Antibodies |
| ☐ | ☒ Eukaryotic cell lines |
| ☒ | ☐ Palaeontology and archaeology |
| ☐ | ☒ Animals and other organisms |
| ☒ | ☐ Clinical data |
| ☒ | ☐ Dual use research of concern |

## Methods

| n/a | Involved in the study |
|---|---|
| ☒ | ☐ ChIP-seq |
| ☐ | ☒ Flow cytometry |
| ☐ | ☒ MRI-based neuroimaging |

# Antibodies

Antibodies used

Antigen conjugate, clone, distributor, catalogue number, LOT number

In vivo antibodies
mCD4, GK1.5, BioXCell, BE0003-1, LOT: 699918O1B
mCD8a, 2.43, BioXCell, BE0061, LOT: 624616A2
mNK1.1, PK136, BioXCell, BE0036, LOT: 735620O1

Immunohistochemistry antibodies
mCD8, PE, 56-6.7, Invitrogen, 12-0081-82, LOT: 2207581

Flow cytometry antibodies
mCD3 BV711, 17A2, BioLegend, 1100241, LOT: B245637
mCD45 BV510, 30-F11, BioLegend, 103138, LOT: B333193
mCD11b PE-dazzle, M1/70, BioLegend, LOT: B316038
mCD3 BV711, 17A2, BioLegend, 1100241, LOT: B245637
mCD4 PE Texas Red, RM4-5, Invitrogen, MCD0417, LOT: 1837385
mCD8 AF700, 53-6.7, BioLegend, 100730, LOT: B326021
mF4/80, BV421, BM8, BioLegend, 123137, LOT: B295586
mMHCII AF700, M5/114.15.2, Biolegend, 107606, LOT: B264454
mPD-L1, BV605, 10F.9G2, BioLegend, 124321, LOT: B245034
mLy6C, FITC, HK1.4, BioLegend, 128006, LOT: B217035
mLy6C, APC, HK1.4, BioLegend, 128016, LOT: B274271
mFoxP3, FITC, FJK-16s, Invitrogen, 11-5773, LOT: 2007700
mLag3, PE-Cy7, eBioC9B7W, Invitrogen, 25-2231-82, LOT: 4284448
mCD45, APC-Cy7, 30-F11, BD Biosciences, 557659, LOT: 9003933
mCD25, PerCPCy5.5, PC61, BioLegend, 102030, LOT: B198863
mPD-L1, BV711, 10F.9G2, BioLegend, 124319, LOT: B309517
mPD-1, BV421, 29F.1.A12, BioLegend, 135218, LOT: B256183
mKi67, eFlour450, SolA15, Invitrogen, 48-5698-82, LOT: 1998365
mNK1.1, Vio Bright B515, REA1162, Miltenyi Biotec, 130-120-503, LOT: 5900109216
CD38, FITC, 90, BioLegend, 102705, LOT: B235499
CD86, PE, GL-1, BioLegend, 105007, LOT: B248265
CD64, PerCP-eFluor 710, X54-5/7.1, Invitrogen, 46-0641-80, LOT: 2161982
MHC II, PE-Cy7, M5/114.15.2, BioLegend, 25-5321-82, LOT: 4332615
MerTK, Brilliant Violet 421, 108928, BD Biosciences, 747837, LOT: 8333892
CD172, APC/Cy7, P84, BioLegend, 144017, LOT: B258707
CD71, Brilliant Violet 510, RI7217, BioLegend, 113823, LOT: B305768
fixable viability dye eFluor780, eBioscience, 65-0865, LOT: 1977883
fixable viability dye eFluor520, eBioscience, 65-0867, LOT: 1915314
CD16/CD32 Monoclonal Antibody, unconjugated,, eBioscienceProduct 11-4811-85

Validation

Validation data of flow cytometry and immunohistochemistry antibodies can be found on the suppliers' website, e.g. in technical data sheets. Antibodies against critical and rare antigens have been titrated previously using positive control cells. In vivo antibodies have been validated repeatedly in previous experiments (Aslan et al. Nat Commun. 2020).

# Eukaryotic cell lines

Policy information about cell lines and Sex and Gender in Research

Cell line source(s)

Murine glioma cell line GL261 was purchased from National Cancer Institute (NCI Tumor Repository)

Authentication

No authentication of GL261 cell line was performed.

| | |
|---|---|
| Mycoplasma contamination | Cell lines were tesed negative for mycoplasma contamination regularly and before in vivo use. Highthroughput Multiplex Cell contamination (McCT), Schmitt M. et al. 2009 |
| Commonly misidentified lines (See ICLAC register) | no commonly misidentified lines from the ICLAC register were used in this study |

## Animals and other research organisms

Policy information about studies involving animals; ARRIVE guidelines recommended for reporting animal research, and Sex and Gender in Research

| | |
|---|---|
| Laboratory animals | C57BL/6J or C57BL/6N wild-type (WT) mice were purchased from Janvier Labs. Female age-matched mice were used for in vivo experiments. All mice were 7-12 weeks of age at use. Mice were kept under SPF conditions at the animal facility of the DKFZ Heidelberg. |
| Wild animals | n/a |
| Reporting on sex | female mice were used in this study. It is expected that animal gender does not influence the results |
| Field-collected samples | n/a |
| Ethics oversight | All animal protocols were performed in compliance with the laboratory animal research guidelines and were approved by the governmental authorities (animal protocols: G27-17 and G35-22, regional administrative authority, Regierungspräsidium Karlsruhe, Germany) |

Note that full information on the approval of the study protocol must also be provided in the manuscript.

## Flow Cytometry

### Plots

Confirm that:

- [x] The axis labels state the marker and fluorochrome used (e.g. CD4-FITC).
- [x] The axis scales are clearly visible. Include numbers along axes only for bottom left plot of group (a 'group' is an analysis of identical markers).
- [x] All plots are contour plots with outliers or pseudocolor plots.
- [x] A numerical value for number of cells or percentage (with statistics) is provided.

### Methodology

| | |
|---|---|
| Sample preparation | Murine Gl261-containing brain hemispheres were excised, washed in HBSS (Sigma-Aldrich) and cut into small pieces before tissue disruption in HBSS supplemented with 50 µg/ml Liberase D for 0,5 h under slow rotation at 37 °C. Dispersed tissue was mashed through a 100µm and 70 !m cell strainer and lymphocytes. For Gl261 sampleas, myelin removal was performed by percoll density gradient as described in online methods. Murine splenocytes were isolated by homogenization using a cell strainer and ACK lysis. In some cases, as described in the online methods, cells were treated with Brefeldin A to prevent secretion of cytokines, chemokines, and other secretory proteins before analysis. Generation of BMDM cultures was performed according to previously established protocols. Briefly, bone marrow cells were flushed from the tibia and femurs of C57BL/6N wild-type mice (8-10 weeks of age) using ice-cold Hanks' Balanced Salt Solution (HBSS) and filtered through a 70 µm cell strainer and plated at a density of 3.5 x 105 cells/ml. Cells were differentiated for one week using RPMI medium supplemented with 10 ng/ml M-CSF (M9170, Sigma-Aldrich), 10 % fetal bovine serum (FBS) and 1 % penicillin/streptomycin (Gibco). |
| Instrument | FACS Canto II , FACS Aria II, LSRFortessa (all BD Biosciences), Attune NxT (Thermo Fisher Scientific), AURORA spectral flow cytometer (Cytek Biosciences) |
| Software | Data was collected with FACS Diva Software (version 9, BD Biosciences) or Attune NxT Software version 2.5. Data was analysed with Flow Jo Version 9 or 10. |
| Cell population abundance | The study did not involve sorting of specific cell populations. |
| Gating strategy | Lymphocytes were defined by size and granularity in FSC-A vs. SSC-A plots. Subsequently, duplets were excluded in FSC-A vs FSC-H plots and dead cells were excluded by means of fixable viability dye positivity. T cells were gated according to the lineage marker CD45 and CD3, then T cell types were gated in CD4 vs CD8 plots. Infiltrating Gl261 CD11b myeloid cells were gated as CD45highCD11b+, microglia as CD45lowCD11b+ of living single cells in CNS samples. Gating strategies of multi-parameter flow cytometry analysis is shown in Extended Data Figures. Boundaries between positive and negative cells were defined by use of fluorescence minus one (FMO) controls. |

- [x] Tick this box to confirm that a figure exemplifying the gating strategy is provided in the Supplementary Information.

# Magnetic resonance imaging

## Experimental design

Design type: Block design. All animals within one experiment were subjected to MRI on the same day

Design specifications: Animals were subjected to MRI 3 times during one experiment at intervals between 6 and 7 days. I.e. MRI was performed on days 13, 19, and 26 post surgery if not otherwise stated in the online methods and figure legends.

Behavioral performance measures: n/a

## Acquisition

Imaging type(s): structural T2 and T1-weighted structural magnetic resonance imaging, diffusion tensor imaging, multi-gradient echo T2* relaxometry

Field strength: 9.4 Tesla small animal MRI

Sequence & imaging parameters: MR imaging included a standard RARE T2-w and T1-w post-Gd-contrast sequence to monitor tumor volume (T2-w parameters: 2D sequence, 0.078 mm in-plane resolution, TE: 33 ms, TR: 2500 ms, flip angle: 90̊ , acquisition matrix: 200 x 150, number of averages: 2, slice thickness: 0.7 mm, duration: 2 min 53 s; T1-w parameters: 2D sequence, 0.1 mm in-plane resolution, TE: 6 ms, 1000 TR: ms, flip angle: 90̊ , acquisition matrix: 256 x 256, number of averages: 2, slice thickness: 0.5 mm, duration: 5 min). Further functional MR imaging included diffusion tensor imaging (parameters: 2D EPI sequence, 30 diffusion gradient directions, 0.125 mm in-plane resolution, TE: 20 ms, TR: 3400 ms, flip angle: 90°, acquisition matrix: 96 x 96, number of averages: 1, slice thickness: 0.7 mm, duration: 7 min 56 s) and multi-gradient echo imaging (MGE parameters: 3D sequence, 0.1 mm in-plane resolution, TE: 2.57 ms, TR: 73.43 ms, flip angle: 20°, acquisition matrix: 200 x 200, number of averages: 2, slice thickness: 0.1 mm, duration: 22 min 7 s). As contrast agent 0.2 mmol/kg Dotarem (Guerbet) was administered i.v. to assess BBB integrity with T1-w. MGE T2* relaxometry was used for macrophage tracking using the ultrasmall superparamagnetic iron oxide (USPIO) nanoparticle ferumoxytol (Feraheme; AMAG Pharmaceuticals Inc.). Imaging was performed before and 24 hours after ferumoxytol (dose of 30mg/kg).

Area of acquisition: whole brain scan

Diffusion MRI: ☒ Used ☐ Not used

Parameters: Single shell 2D EPI-DTI was acquired with 30 diffusion gradient directions (DTI Spinecho EPI, 4 Segments, Double Sampling, FOV 12mm x 15mm, Matrix 96 x 128, Partial Fourier Factor 1.5, 17 Slices, Slice Thickness 0.7mm, TE 20ms, TR 3400ms, Fat Suppression, FOV Saturation, Bandwith 333333Hz, Diffusion encoding: Gradient duration 3 ms, Gradient Separation 9 ms, Bvalue 1500 s/mm$^2$). Cardiac-gating was not applied.

## Preprocessing

Preprocessing software: MR Images were exported as DICOM files and preprocessed in nordicICE Software (v4.2.0). For the quantification of MRI diffusion data, FA (fractional anisotropy) of the tumor core was segmented manually. The ratio of normal brain tissue and the tumor diffusion FA was calculated.

Normalization: MRI data were neither normalized for tumor volumetry performed on T2w-images nor for analysis of FA-values in the tumor ROI. The calculation of T2* relaxation times included noise filtering. Sigma was calculated for the whole dataset and voxels with a deviation larger than 4 sigma were removed. Radiomics analysis included brain extraction and Z-score normalization of T2w-datasets.

Normalization template: Data were not normalized to a standard space.

Noise and artifact removal: The calculation of T2* relaxation times included noise filtering. Sigma was calculated for the whole dataset and voxels with a deviation larger than 4 sigma were removed. All other data were not processed for noise or artifact removal.

Volume censoring: MR images were exported as DICOM files and were visualized in OsiriX Imaging software (version 4.12; Pixmeo) and FIJI (FIJI ImageJ, Version 1.52). For the quantification of MRI data, tumor volumes were segmented semi-automatically using AMIRA (FEI). Volumes were exported to Microsoft Excel. No censoring was performed.

## Statistical modeling & inference

Model type and settings: Machine-learning-Gradient boosting in R version 4.0.3 (R Foundation for Statistical Computing, Vienna, Austria) using the caret library. Tuning parameters (boosting iterations, max tree depth, shrinkage and min. terminal node size) were automatically optimized via resampling procedures.

Effect(s) tested: Predictive modeling of treatment failure at MRI3 from imaging data of previous timepoints.

Specify type of analysis: ☐ Whole brain ☒ ROI-based ☐ Both

Anatomical location(s) | Lesion volumes (MRI1 baseline lesion volumes, MRI2 during treatment lesion volumes, and MRI3 post treatment lesion volumes) were segmented on T2-w MR images using a fully automated artificial neural network from the nnUNET framework and trained on the data from Aslan et al.

Statistic type for inference (See Eklund et al. 2016) | Radiomic data was analysed up to a voxel basis.

Correction | n/a

## Models & analysis

| n/a | Involved in the study |
|---|---|
| ☒ | ☐ Functional and/or effective connectivity |
| ☒ | ☐ Graph analysis |
| ☐ | ☒ Multivariate modeling or predictive analysis |

Multivariate modeling and predictive analysis

Radiomic features were calculated from the T2-hyperintense tumor volume from the first MRI and the change in features between the first and second MRI for radiomic signature discovery. Based on these radiomic features (n=383 from each time point), gradient boosting machine-learning models were constructed to predict treatment failure at the third MRI. Model performance was evaluated using ten-fold cross validation with 10x repeats. Lesion volumes (MRI1 baseline lesion volumes, MRI2 during treatment lesion volumes, and MRI3 post treatment lesion volumes) were segmented on T2-w MR images using a fully automated artificial neural network from the nnUNET framework and trained on the data from a separate project (Aslan et al., Nat Communications, 2020). Segmentations were visually checked for consistency, MRI images were skull stripped and normalized using Z-score normalization, and radiomic features were calculated from the tumor segmentation masks from T2-w MR images for each mouse from both time points using the medical imaging interaction toolkit (MITK, www.mitk.org). Images were skull stripped and Z-score normalized using FSL (FMRIB, Oxford). A radiomic feature set consisting of all features from MRI1 as well as the absolute difference in each radiomic feature between time points MRI1 and MRI2 was used as an input for predictive modeling of treatment failure (i.e., prediction of response yes vs no) at MRI3 (implemented in R version 4.0.3 with the caret library. Predictive modeling was performed using a gradient boosting machine-learning algorithm that iteratively constructs an ensemble of weak decision tree learners through boosting to form a single strong predictive model (the tuning parameters (boosting iterations, max tree depth, shrinkage, and min. terminal node size) were automatically optimized via resampling procedures). The performance of the gradient boosting classifier was assessed based on a ten-times repeated ten-fold cross validation resampling procedure. The held-out predictions in each of the resampling iterations were used to calculate the accuracy, area under the receiver operating characteristic (ROC), sensitivity, specificity, no information rate (largest class percentage for each molecular parameter, i.e., the prediction or accuracy by chance), and a hypothesis test (using the binom.test function) to evaluate whether the accuracy rate is greater than the no information rate. P-values<0.05 were considered significant.
